# Intervention treatment reducing cellular senescence inhibits tubulointerstitial fibrosis in diabetic mice following acute kidney injury

**DOI:** 10.1042/CS20231698

**Published:** 2024-03-05

**Authors:** Gregory H. Tesch, Frank Y. Ma, Elyce Ozols, David J. Nikolic-Paterson

**Affiliations:** 1 Department of Nephrology, Monash Medical Centre, Clayton, Victoria, Australia; 2 Centre for Inflammatory Diseases, Monash University, Clayton, Victoria, Australia

**Keywords:** acute kidney injury, ASK1 inhibitor, cellular senescence, diabetic kidney disease, HSP90 inhibitor, tubulointerstitial fibrosis

## Abstract

Senescence of kidney tubules leads to tubulointerstitial fibrosis (TIF). Proximal tubular epithelial cells undergo stress-induced senescence during diabetes and episodes of acute kidney injury (AKI), and combining these injuries promotes the progression of diabetic kidney disease (DKD). Since TIF is crucial to progression of DKD, we examined the therapeutic potential of targeting senescence with a senolytic drug (HSP90 inhibitor) and/or a senostatic drug (ASK1 inhibitor) in a model of TIF in which AKI is superimposed on diabetes. After 8 weeks of streptozotocin-induced diabetes, mice underwent bilateral clamping of renal pedicles to induce mild AKI, followed by 28 days of reperfusion. Groups of mice (*n*=10–12) received either vehicle, HSP90 inhibitor (alvespimycin), ASK1 inhibitor (GS-444217), or both treatments. Vehicle-treated mice displayed tubular injury at day 3 and extensive tubular cell senescence at day 10, which remained unresolved at day 28. Markers of senescence (Cdkn1a and Cdkn2a), inflammation (*Cd68, Tnf,* and *Ccl2*), and TIF (*Col1a1, Col4a3, α-Sma/Acta2,* and *Tgfb1*) were elevated at day 28, coinciding with renal function impairment. Treatment with alvespimycin alone reduced kidney senescence and levels of *Col1a1*, *Acta2*, *Tgfb1*, and *Cd68*; however, further treatment with GS-444217 also reduced *Col4a3*, *Tnf, Ccl2,* and renal function impairment. Senolytic therapy can inhibit TIF during DKD, but its effectiveness can be improved by follow-up treatment with a senostatic inhibitor, which has important implications for treating progressive DKD.

## Introduction

Amongst individuals with diabetes, about one third will have kidney disease, and a third of this group will develop progressive life-threatening chronic kidney disease (CKD) [1]. Several clinical studies have attempted to identify the underlying factors responsible for the progression of diabetic kidney disease (DKD) and to predict at risk patients using panels of biomarkers [2]; however, these causes remain unclear. Oxidative stress, DNA damage, macrophage-mediated inflammation, tubular senescence and kidney fibrosis are characteristic features of progressive DKD [3]. However, current therapeutic options don’t specifically target any of these disease mechanisms. Diabetes induces kidney oxidative stress and DNA damage, thereby promoting senescence of tubular epithelial cells (TECs), which leads to hyperglycemic memory, chronic inflammation and tubulointerstitial fibrosis (TIF) [4,5]. Therefore, tubular senescence appears central to the progression of DKD, and is a key therapeutic target for intervention.

Both DKD, and acute kidney injury (AKI) caused by ischemia, involve senescence and damage to kidney tubules, which is associated with inflammation and fibrosis [4]. While healthy kidneys can recover quickly from a moderate ischemic insult, diabetic kidneys are more sensitive to ischemic injury and develop an increased and prolonged tubular injury, leading to maladaptive repair, chronic inflammation and TIF [6,7]. Consequently, it is proposed that progression of DKD, and particularly TIF, occurs after episodes of ischemia [8,9]. The clinical relevance of this concept is supported by a study of >9000 patients showing that the incidence and frequency of episodes of AKI in diabetes patients correlates with progression to CKD and end-stage renal disease [10].

Recent studies have indicated that senotherapies targeting the development, maintenance and products of cellular senescence have potential for reducing the progression of CKD [11]. Current senotherapy strategies aim to either remove senescent cells from tissues (senolytic agents) or prevent the formation of senescent cells and their secretory products (senostatic agents). Both these strategies have been shown to be effective in reducing the impact of cellular senescence on kidney injury.

Heat shock proteins (HSPs) are a family of intracellular chaperones with diverse functions. They are mostly associated with enabling protein folding under stress conditions, and promoting cell cycle and repair processes. One member, HSP90, has been shown to protect senescent cells from undergoing apoptosis by facilitating phosphorylation of its client protein, AKT [12]. In contrast, inhibitors of HSP90 selectively deplete senescent cells, which is thought to occur by destabilizing the kinases AKT and ERK which allows apoptosis to occur [13,14]. One study has shown that treatment with a HSP90 inhibitor (alvespimycin) for one week reduces the senescence marker p16^INK4a^/Cdkn2a by 60% in mouse kidneys with accelerated ageing (progeroid syndrome) [13]. Other studies have also show that alvespimycin can reduce kidney injury in animal models of acute and chronic kidney injury which involve oxidative stress [15,16]. Therefore, alvespimycin has significant potential as a senolytic agent in CKD.

Current senostatic agents include therapies targeting NF-kB, p38 MAPK, JNK, GATA4, mTOR, BRD4, and cGAS/STING [17,18]. Increased p38 MAPK and JNK signaling is a common feature of acute and CKDs, which can be induced by hyperglycemia and pathological levels of oxidative stress via upstream activation of apoptosis signal-regulating kinase-1 (ASK1) [19]. The active (phosphorylated) forms of p38 MAPK and JNK are markedly increased in epithelial cells in injured kidneys [20–22], and inhibition of these kinases reduces kidney fibrosis in disease [21,23]. Notably, p38 MAPK can phosphorylate p53, Cdkn1a, and Cdkn2a; thereby inhibiting cell cycle progression and promoting senescence [24–26]. In addition, inhibition of p38 MAPK or ASK1 reduces production and secretion of senescence-associated secretory proteins (SASPs) by cultured cells [20,26]. Hence, targeting of ASK1-p38 MAPK signaling has been postulated to be a senostatic strategy for inhibiting kidney damage induced by cellular senescence. This concept is supported by animal model studies showing that and ASK1 inhibition reduces kidney injury associated with diabetes or ischemic injury, as well as clinical trials demonstrating that ASK1 inhibitor protects patients from DKD [19].

In the present study, we examined whether intervention with a senolytic therapy (a HSP90 inhibitor), a senostatic therapy (an ASK1 inhibitor), or combined treatments would provide effective protection against the development of CKD caused by ongoing diabetes and acute ischemic injury.

## Materials and methods

### Animals

Male inbred C57BL/6J mice were sourced from the Monash Animal Research Platform (Clayton, Victoria, Australia). All animal experiments were performed at the Monash Medical Centre Animal Facility. The Monash Medical Centre Animal Ethics Committee gave approval for these studies, and they conformed with the 8th Edition of the Australian Code of Practice for the Care and Use of Animals for Scientific Purposes.

### Induction and assessment of Type 1 diabetes

Type 1 diabetes was induced in 10- to 12-week-old male C57BL6/J mice using multiple low doses of streptozotocin (STZ). On each of five consecutive days, mice were fasted for 6 h and then given an intraperitoneal injection of 55 mg/kg STZ freshly dissolved in sodium citrate buffer (pH 4.5). Blood glucose was measured after a 3 h fast by weekly tail vein sampling (Stat Strip Xpress, Nova Biomedical, Whaltham, MA, U.S.A.) after the STZ injections. Mice achieving a consistent fasting blood glucose level of >16 mmol/L at 2 weeks after STZ were considered diabetic. After becoming diabetic, fasting blood glucose levels were maintained within the target range (20–28 mmol/L), with 0.4U of long-acting insulin (Protophane, Novo Nordisk, Baulkham, Australia) given by subcutaneous injection as required. Plasma was collected from the tail vein to measure HbA1c (DCA Vantage Analyzer, Siemens Healthcare Diagnostics, Tarrytown, NY, U.S.A.) prior to IRI surgery (week 8) and at the end of the study (week 12). Diabetic mice with an HbA1c >8% at week 8, were used for sham or ischemia–reperfusion injury (IRI) surgery and randomly assigned to treatment groups.

### Ischemia–reperfusion injury (IRI) surgical procedure

Diabetic mice were anesthetized with ketamine and xylazine and body temperature was maintained at 37 degrees using a rectal thermometer linked to heating blanket (Homeothermic monitor system, Harvard Apparatus, Holliston, MA, U.S.A.). After a midline abdominal incision, the right and then the left renal pedicle were clamped using non-traumatic vascular clamps (B-1A microvascular clamp for arteries (00397), S&T Microsurgical Instruments, Birmingham, AL, U.S.A.) and the abdomen closed with a temporary suture to minimize fluid loss. Clamps were then removed from the right and then the left renal pedicle giving each kidney exactly 12 min of clamping (ischemia), with reperfusion of each kidney monitored visually [6,7]. Next, the abdomen was sutured in two layers using 5-0 suture, and analgesia provided by 2–3 drops of bupivacaine onto the sutures and a subcutaneous injection of 0.05 mg/kg buprenorphine. Fluid management was provided by subcutaneous saline injection (1 ml spread over several sites) at the time of surgery and at 4 h after surgery. In addition, mice were given mouse chow mashed together with liquid Ensure (Abbot Laboratories, Chicago, IL, U.S.A.) in a Petri dish on the cage floor for the first 2 days after surgery, and cages were kept on a heating pad overnight after surgery. Sham-operated diabetic mice underwent the same procedure, but without clamping of the renal pedicles.

### Sample size and power calculations

Sample size was estimated using the MINITAB power curve for one-way ANOVA. Our prime aim compares three different treatments to vehicle alone in diabetic mice at day 28 after IRI. For our main endpoint (fibrosis), we aimed to detect a difference in means of ≥20% with an expected SD of 12% (*α* is 0.05). Our power calculation showed that this would be achieved with 95% power in groups of 12 mice. Smaller groups of mice (*n*=10–11) were used for sham-operated diabetic mice, and mice with diabetes and IRI examined at earlier timepoints after IRI (day 3 or 10).

### Experimental design and treatments

At 8 weeks after STZ injections, mice with equivalent diabetes underwent 12 min of bilateral clamping of the renal pedicles (as described above) to induce IRI. These diabetic IRI mice were assigned to groups (*n*=10–12) receiving treatment or vehicle, which were killed on days 3, 10 or 28 after IRI surgery. To deplete senescent cells, diabetic IRI mice were given a senolytic HSP90 inhibitor (Alvespimycin – Med Chem Express, 10 mg/kg in normal saline [13]) by intraperitoneal injection on days 3, 6 and 9 after IR surgery and groups were killed on days 10 or 28. To prevent new senescent cells forming and suppress production of senescent-associated secretory proteins (SASPs), diabetic IRI mice were given a senostatic ASK1 inhibitor (GS-444217 – Gilead Sciences, 30 mg/kg/bid [20]) in vehicle (75% propylene glycol: 10% solutol HS15: 10% water: 5% ethanol) by oral gavage from day 10 to 28 post IR. To assess combined senolytic and senostatic treatment, diabetic IRI mice were given HSP90 inhibitor (days 3, 6 and 9) followed by ASK1 inhibitor (days 10–28). Disease control groups consisted of diabetic IRI mice receiving no treatment (day 3) or vehicle alone (days 10 and 28). Groups of age-matched non-diabetic mice (*n*=12) and sham-operated diabetic mice (*n*=10) were used as controls. At the end of experimentation, mice were given intraperitoneal injections of ketamine and xylazine to induce full anesthesia and were then killed by exsanguination followed by cervical dislocation.

### Biochemistry

Urine samples were collected from age-matched mice (diabetic IR, diabetic sham, non-diabetic) placed in metabolic cages for 6 h on the day before killing. Plasma was obtained from heparinised whole blood collected by cardiac puncture of anesthetized mice at the time of killing. Urine levels of albumin and CDKN1A were measured by ELISA using matched antibody pair kits (Abcam, Oxford, U.K.). For detection of CDKN1A, some urine samples were concentrated 10-fold by centrifugation using polyethersulfone protein concentrators with a 3KDa molecular-weight cut-off (Pierce Biotechnology, Rockford, IL, U.S.A.). Blood urea nitrogen (BUN) and creatinine levels in urine and plasma were measured using a Dupont ARL Analyser by the Department of Biochemistry, Monash Health.

### Antibodies

The primary antibodies used in the present study were: rabbit anti-Cdkn1a (EPR18021 at 1:500 dilution; Abcam, Cambridge, U.K.), rabbit anti-α-SMA (EPR5368, 1 in 8000 dilution; Abcam), rabbit anti-collagen type I (E8F4L, 1 in 400 dilution; Cell Signaling Technology, Danvers, MA, U.S.A.), or goat anti-KIM-1 (AF1817 at 1:500 dilution, R&D systems, Minneapolis, MN, U.S.A.). Irrelevant rabbit and goat primary antibodies were used as controls to ensure the specificity of immunostaining.

### Immunohistochemistry

For immunostaining of Cdkn1a, α-SMA, collagen I, and KIM-1, 4 μm sections of kidney fixed in either formalin or methacarn were deparaffinised and rehydrated through graded ethanol into PBS. Heat antigen retrieval (95°C, 40 min) was required for antibody detection of p21 (using Tris-EDTA, pH 9) and collagen I and KIM-1 (using sodium citrate, pH 6). After blocking with 5% bovine serum albumin (BSA) at room temperature for 30 min, sections were incubated overnight at 4°C with primary antibodies diluted with 5% mouse serum and 5% rabbit or sheep serum (depending on the species of the primary antibody). PBS washing was performed after antibody incubation and each subsequent step. Endogenous peroxidase was blocked in 3% H_2_O_2_ in distilled water for 10 mins. Endogenous biotin was blocked with an avidin/biotin blocking kit (SP-2001; Vector Laboratories, Burlingame, CA, U.S.A.). Sections were then incubated with biotinylated secondary antibodies (1:500 dilution) against rat IgG (BA-400; Vector), rabbit IgG (65-6140; Invitrogen, Waltham, MA, U.S.A.), or goat IgG (A105-18; Life Technologies, Carlsbad, CA, U.S.A.) for 45 min. Thereafter, sections were incubated with ABC peroxidase (PK-6100; Vector) for 45 min and then either diaminobenzidine (DAB, SK-4100; Vector) to produce a brown product or Vector SG (SK-4700; Vector) to produce a blue-gray. After color development, stained sections were dehydrated through graded ethanol into histolene, and mounted with Eukitt mounting medium (Sigma Aldrich, Melbourne, Australia).

### Analysis of immunohistochemistry

Immunohistochemistry images were obtained using an Olympus BX43 microscope and an Olympus DP27 camera, and were analyzed with Olympus CellSens Standard 1.18 software (Olympus LifeSciences, Japan). For analyses, images were collected at 100× magnification for the entire kidney cortex on the section. The region of interest (renal interstitium) was manually defined to exclude glomeruli and large vessels, and image analysis was performed following adjustment of threshold settings. Cells immunostained for Cdkn1a were counted/mm^2^ of the kidney cortical interstitium. Immunostaining for α-smooth muscle actin and collagen 1 was scored as the percentage area stained within the kidney cortex, excluding glomeruli and large vessels. All slides and images were blinded at time of image analysis.

### Real-time RT-PCR

Snap-frozen kidney tissue (50–100 mg) was added to TRIzol solution (Invitrogen), homogenised, and total RNA was extracted using the RiboPure RNA purification kit (Invitrogen), according to the manufacturer’s protocol. Total RNA (7 μg) per sample was reversed transcribed into cDNA using the Superscript III First-Strand Synthesis kit (Invitrogen), according to the manufacturer’s protocol. Real-time PCR was performed using a StepOne Real-Time PCR System (Applied Biosystems, Waltham, MA, U.S.A.) and a total reaction volume of 20 μl containing cDNA, Taqman Gene Expression Master Mix (Applied Biosystems), and Taqman gene expression assays for the gene of interest (Supplementary Table S1) and the *Actb* internal control (4352341E, Applied Biosystems). Thermal cycling conditions were: 37°C for 10 min, 95°C for 5 min, followed by 50 cycles of 95°C for 15 s, 60°C for 20 s and 68°C for 20 s. The relative amount of cDNA was calculated using comparative threshold cycle (ΔΔCt) method.

### Statistics

Statistical differences were analyzed by either the unpaired Student’s *t-*test or one-way ANOVA with Tukey’s or Dunnet’s multiple comparison post-test. Data were recorded as mean ± 1 SD (unless otherwise stated), with *P*<0.05 considered significant. All analyses were performed using GraphPad Prism 9.0 (GraphPad software, San Diego, CA, U.S.A.).

## Results

### Development of diabetes in experimental mice

**Figure 1:**
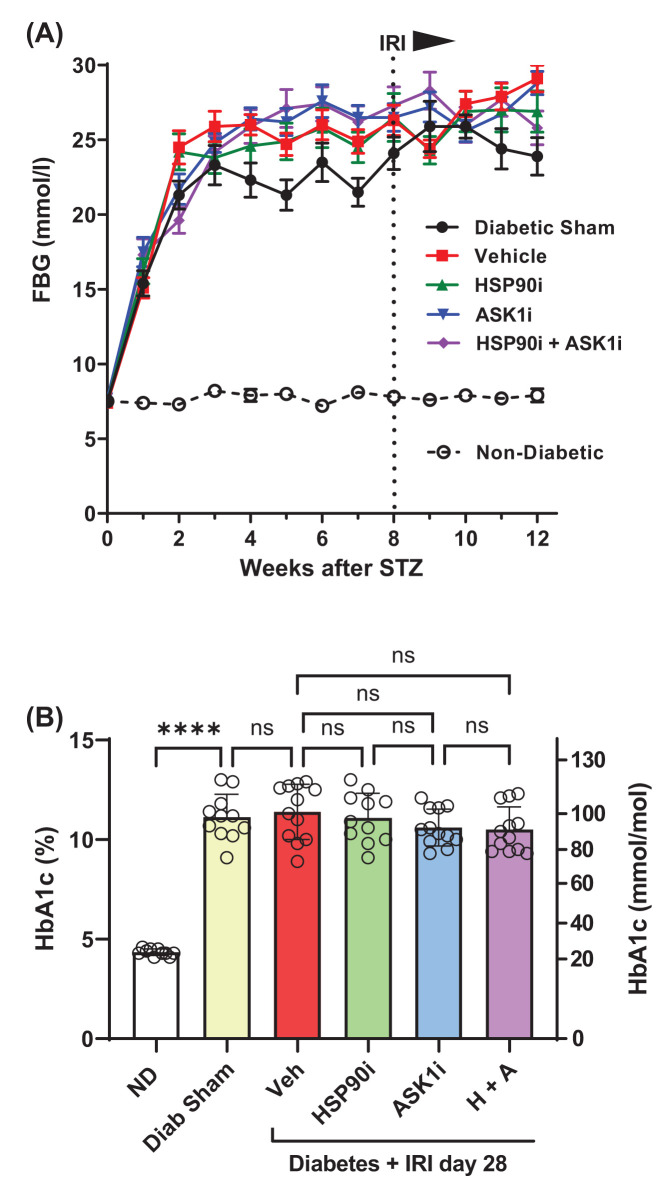
Development of diabetes in experimental mice At 8 weeks after receiving streptozotocin (STZ), groups of diabetic mice were given IRI surgery to induce AKI. These mice were then treated with either vehicle (Veh, days 3–28), alvespimycin (HSP90i, days 3–9), GS-444217 (ASK1i, days 10–28), or alvespimycin followed by GS-444217 (H + A). Age-matched non-diabetic mice (ND) and sham-operated diabetic mice (Diab Sham) were used as controls. The development of diabetes was monitored by measurements of (**A**) fasting blood glucose (FBG) performed weekly and (**B**) the accumulation of glycated hemoglobin (HbA_1c_ levels) in blood at week 12 after STZ. Data = mean ± SEM in (A) and mean ± SD in (B); *n*=10–12; ****P<0.0001; ns = non-significant.

Experimental mice showed a progressive rise in fasting blood glucose levels during the first 2 weeks after streptozotocin, which reached a plateau thereafter (Figure 1A). IRI surgery at week 8, or the subsequent use of intervention therapies (alvespimycin, GS-444217 or combined treatment) had no impact on the elevated fasting blood glucose levels in diabetic mice (Figure 1A). The development of diabetes resulted in a marked increase in the levels of glycated hemoglobin (HbA_1c_) in mice at week 12 after streptozotocin, which was equivalent in all diabetic groups and unaffected by any interventions used (Figure 1B). Over 12 weeks, non-diabetic control mice showed a 23% increase in mean body weight, whereas the body weight of diabetic mice remained unchanged and unaffected by IRI or treatments (Supplementary Figure S1)

### IRI induces chronic tubular damage in diabetic mice

**Figure 2:**
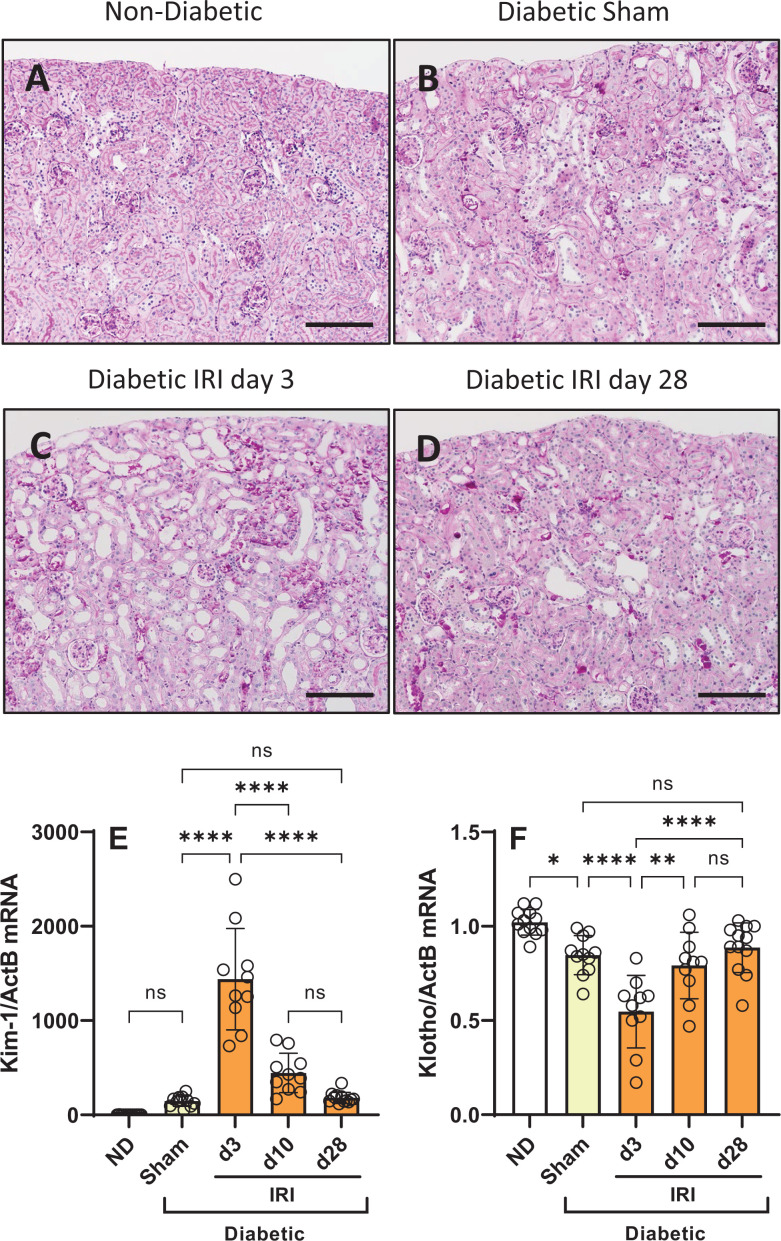
Tubular injury in diabetic mice following IRI Histological staining with PAS shows: (**A**) normal kidney structure in a non-diabetic mouse; (**B**) mild tubular injury (dilation, atrophy) and tubular glycogen deposits in a diabetic mouse at day 28 after sham surgery; (**C**) substantial tubular injury (dilation, atrophy, and cell loss) and tubular glycogen deposits in a diabetic mouse after 3 days of IRI; and (**D**) reduced numbers of tubules displaying histological damage in diabetic mice at day 28 after IRI. Images (A**–**D) Bar = 200 µm. Further gene expression analysis of whole kidneys found that (**E**) *Kim-1* was increased with tubular injury and (**F**) *Klotho* was decreased with tubular injury. Data = mean ± SD; *n*=10–12; ****P<0.0001, **P<0.01, *P<0.05; ns = non-significant.

Compared with non-diabetic mouse kidneys with normal tubular structure (Figure 2A), the kidneys of diabetic mice had some cortical tubules that were dilated and had accumulated glycogen, as indicated by pink PAS deposits in tubules (Figure 2B). Tubular damage (dilation, atrophy, and cell loss) was most prominent in the proximal tubules of the kidney cortex and was substantially worse in diabetic mice at day 3 after IRI (Figure 2C). There was also some damage to medullary tubules at day 3 after IRI, which was mostly confined to the outer medulla (not shown) and was no longer evident a day 10. In comparison, proximal tubular damage was reduced but still persisted at day 28 after IRI (Figure 2D). Evidence of mild tubular injury in mice with diabetes and sham IR surgery was demonstrated by an increase in *Kim-1* gene expression and a decrease in *Klotho* gene expression compared with non-diabetic controls (Figure 2E,F). Applying IRI to diabetic mice resulted in a 10-fold further increase in *Kim-1* gene expression at day 3, which resolved back to pre-IR injury levels by day 28 (Figure 2E). In contrast, *Klotho* gene expression was reduced 50% in diabetic mice at day 3 after IRI but returned to pre-IRI levels at day 28 (Figure 2F).

### IRI exacerbates tubular senescence in diabetic mice, which remains chronically elevated

**Figure 3:**
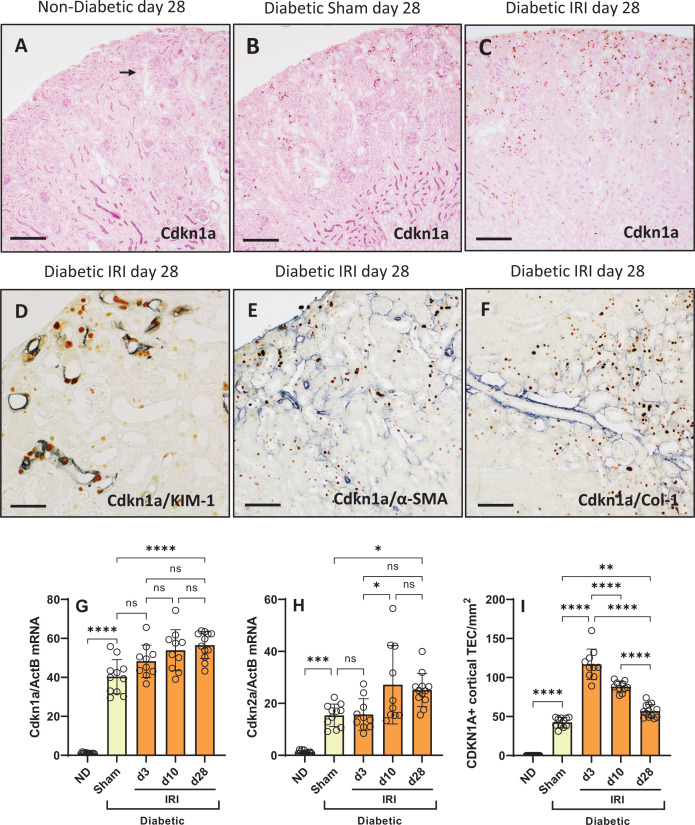
Impact of IRI on tubular senescence in diabetic mice Immunostaining shows that CDKN1A+ cells (displayed as brown nuclei) are: (**A**) rarely detected in a normal non-diabetic mouse kidney (arrow); (**B**) frequently detected in mouse kidney tubules after 12 weeks of diabetes; and (**C**) are further increased in the kidney tubules of diabetic mice at day 3 after IRI. Double immunostaining at day 28 after IRI, shows that CDKN1A+ tubular cells (brown nuclei) are associated with: (**D**) KIM-1+ staining (blue-grey) in tubules; (**E**) tubulointerstitial staining of α-smooth muscle actin + myofibroblasts (blue-gray); and (**F**) tubulointerstitial staining of collagen I (blue-gray). Graphs showing kidney gene expression of (**G**) Cdkn1a and (**H**) Cdkn2a indicate that tubular senescence develops in response to diabetes (yellow bar) and is made worse by IRI (orange bars). A graph of CDKN1A+ tubular cells (**I**) shows that senescent tubular cells are present diabetic kidneys (yellow bar) and increase acutely after IRI, followed by a partial reduction during IRI recovery. For panels (A,B,C,E,F), bar = 200 µm; for panel (D), bar = 100 µm. Data (G–I) = individual data points with mean ± SD; *n*=10–12; ****P<0.0001, ***P<0.001, **P<0.01, *P<0.05; ns = non-significant.

Senescence in TECs was identified by nuclear staining of Cdkn1a [27], which was extremely rare in normal mouse kidneys, but was a significant feature in the cortex of mouse kidneys after development of diabetes, which was further increased by IRI (Figure 3A–C). Virtually all kidney senescent Cdkn1a+ cells were TECs that were predominantly in the proximal tubules; however, a small portion of medullary tubules were also found to contain senescent cells. Double immunostaining of diabetic IRI mice showed that many Cdkn1a+ senescent TEC also expressed KIM-1 (a marker of proximal tubular injury) and were in areas with an accumulation of α-smooth muscle actin and collagen I (Figure 3D–F), demonstrating a link between TEC senescence and tubular injury or fibrosis. Compared with normal kidneys, diabetic kidneys displayed increases in gene expression of *Cdkn1a* (40-fold) and *Cdkn2a* (15-fold) at day 28 after sham surgery, which coincided with a 500-fold increase in the number of TECs detected by immunostaining for Cdkn1a (Figure 3G,H). Applying IRI to diabetic mice resulted in a further increase in the gene expression of *Cdkn1a* and *Cdkn2a* at day 10, which was maintained at day 28 (Figure 3G,H). In comparison, immunostaining identified a 3-fold increase in Cdkn1a+ TEC in diabetic mouse kidneys at day 3 after IRI, which gradually declined but remained elevated at day 28 compared with diabetic sham mice (Figure 3I). Similarly, urine excretion of Cdkn1a was acutely elevated at day 3 after IRI (1869 ± 1140 pg/g creatinine, 35-fold↑) compared with diabetic sham mice (63 ± 16 pg/g creatine), but this increase was substantially reduced by day 28 (110 ± 39 pg/g creatinine).

### Senotherapy reduces tubular senescence in diabetic mice with IRI

**Figure 4:**
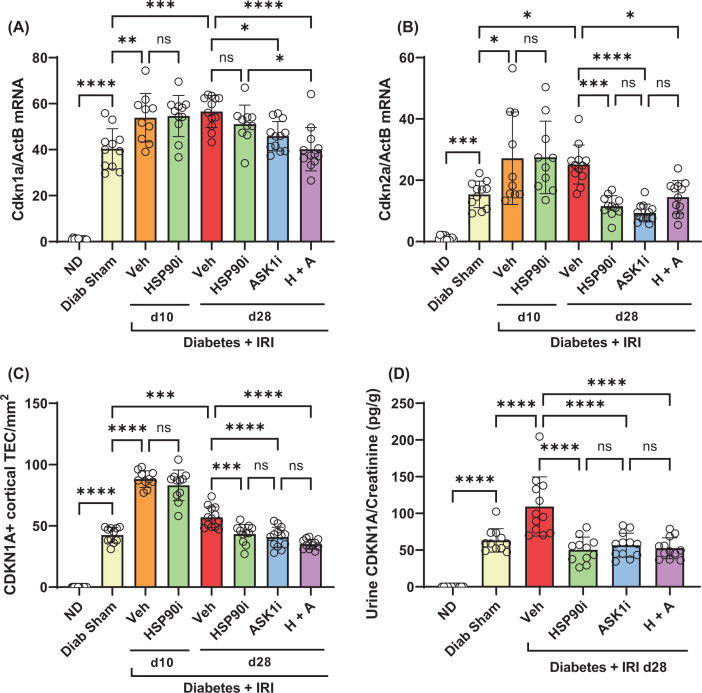
Senotherapies reduce tubular senescence in diabetic mice with IRI Analysis of diabetic mice at days 10 and 28 after IRI identified the impact of alvespimycin (HSP90i, green bars), GS-444217 (ASK1i, blue bars), and combined therapy (H+A, purple bars) on kidney gene expression of (**A**) *Cdkn1a* and (**B**) *Cdkn2a*, (**C**) immunostaining of CDKN1A+ tubular cells, and (**D**) urine excretion of CDKN1A. Non-diabetic mice (ND, white bars), sham-operated diabetic mice (Diab Sham, yellow bars) and vehicle-treated mice with diabetes and IRI (Veh, red bars) were used as controls. Data = individual data points with mean ± SD; *n*=10–12; ****P<0.0001, ***P<0.001, **P<0.01, *P<0.05; ns = non-significant.

Alvespimicyin (an HSP90 inhibitor) was administered to diabetic mice on days 3, 6 and 9 after IRI at a dose previously reported to be senolytic [13]. We established that this dosage of alvespimycin had no impact on liver function at day 10 or day 28 after IRI (Supplementary Figure S2). Compared with vehicle, alvespimycin therapy did not reduce kidney gene expression of *Cdkn1a* or *Cdkn2a*, or the number of Cdkn1a+ TEC, in diabetic kidneys at day 10 after IRI (Figure 4A–C). However, alvespimycin treatment did reduce the gene expression of *Cdkn2a* and the number of Cdkn1a+ TEC in diabetic kidneys at day 28 after IRI (Figure 4A–C), suggesting a delayed response to this senolytic therapy. This protection was further supported by the finding that alvespimycin treatment reduced urine excretion of Cdkn1a at day 28 after IRI (Figure 4D).

GS-444217 (an ASK1 inhibitor) was administered as a senostatic therapy to diabetic mice between days 10 and 28 after IRI. Compared with vehicle, GS-444217 treatment reduced gene expression of *Cdkn1a* and *Cdkn2a*, and the number of Cdkn1a+ TEC, in diabetic kidneys at day 28 after IRI (Figure 4A–C). GS-444217 also reduced the urine excretion of Cdkn1a by these diabetic mice at day 28 after IRI (Figure 4D). Combining alvespimycin and GS-444217 as sequential treatments had a similar effect to GS-444217 alone in terms of reducing these markers of cellular senescence (Figure 4A–D).

### Senotherapy inhibits renal function impairment in diabetic mice after IRI

**Figure 5:**
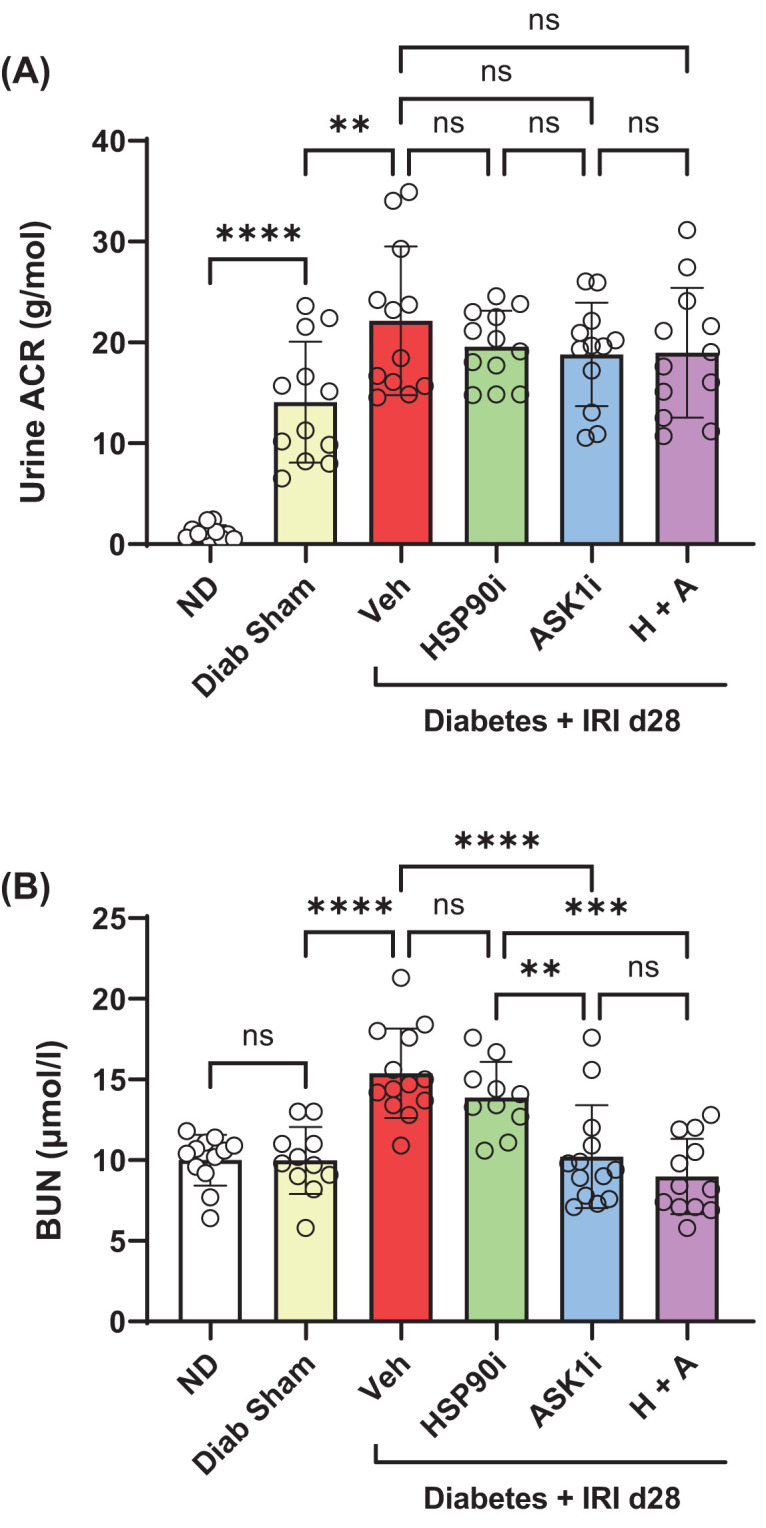
Senotherapy inhibits renal function impairment in diabetic mice with IRI Biochemical analysis of (**A**) the urine albumin creatinine ratio (ACR) and (**B**) blood urea nitrogen was assessed in diabetic IRI mice which received vehicle (Veh, red bars), alvespimycin (HSP90i, green bars), GS-444217 (ASK1i, blue bars), and combined therapy (H+A, purple bars) at day 28 after IRI. Non-diabetic mice (ND, white bars), sham-operated diabetic mice (Diab Sham, yellow bars) were used as controls. Data = individual data points with mean ± SD; *n*=10–12; ****P<0.0001, ***P<0.001, **P<0.01; ns = non-significant.

Compared with normal mice, urine albumin excretion increased 12-fold by day 28 in diabetic sham controls (Figure 5A). Performing IRI on diabetic mice increased urine albumin excretion by a further 80% after 28 days (Figure 5A). In comparison, diabetic IRI mice which received alvespimycin, GS-444217 or combined therapy showed no difference in urine albumin excretion at day 28 compared with vehicle treated mice (Figure 5A).

Blood urea nitrogen (BUN) was used as a measure of renal function because ASK1 inhibitor treatment has been shown to inhibit renal excretion of creatinine, which disappears after 4 weeks in diabetic patients [28]. Levels of BUN were found to be similar in non-diabetic and diabetic sham controls (Figure 5B). In comparison, performing IRI on diabetic mice resulted in a 50% increase in BUN levels at day 28, which was reduced to normal levels by treatment with GS-444217 alone, or alvespimycin and GS-444217, but not alvespimycin alone (Figure 5B).

### Senotherapy suppresses inflammation in diabetic mice after IRI

**Figure 6:**
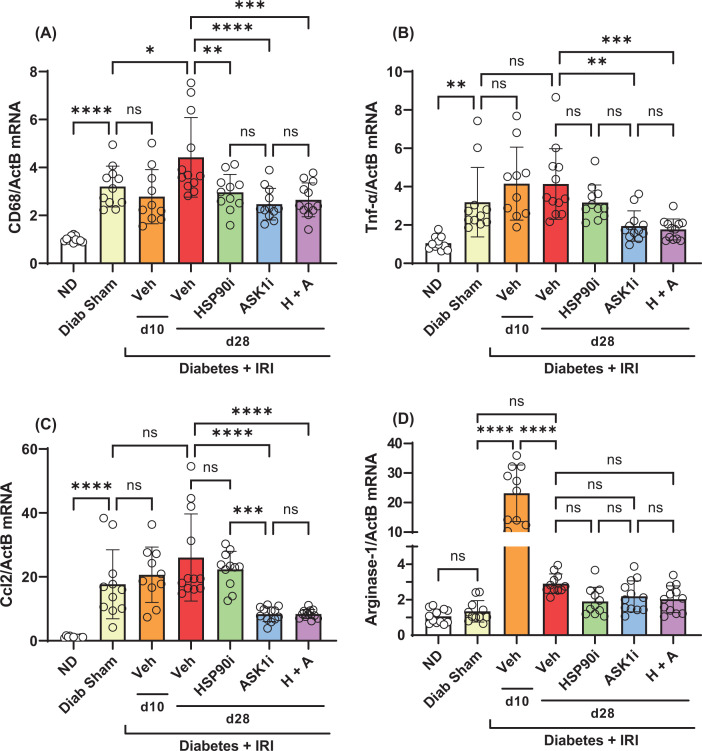
Senotherapies suppress inflammation in diabetic mice with IRI Analysis of diabetic mouse kidneys at days 10 and 28 after IRI identified the impact of alvespimycin (HSP90i, green bars), GS-444217 (ASK1i, blue bars), and combined therapy (H+A, purple bars) on kidney gene expression of (**A**) *Cd68* and (**B**) *Tnf-α*, (**C**) *Ccl2*, and (**D**) *Arginase-1*. Non-diabetic mice (ND, white bars), sham-operated diabetic mice (Diab Sham, yellow bars) and vehicle-treated mice with diabetes and IRI (Veh, red bars) were used as controls. Data = individual data points with mean ± SD; *n*=10–12; ****P<0.0001, ***P<0.001, **P<0.01, *P<0.05; ns = non-significant.

At day 28 in diabetic sham controls, we observed a 3-fold increase in the kidney gene expression of *Cd68* and *Tnf*, and a 17-fold increase in kidney gene expression of *Ccl2* (Figure 6A–C). Performing IRI in diabetic mice did not further increase the kidney expression of these proinflammatory genes at day 28, which remained at similar levels. Compared with vehicle alone, treatment with alvespimycin alone reduced gene expression of *Cd68*, but not *Tnf* or *Ccl2*, at day 28 (Figure 6A–C). However, treatment with GS-444217 alone, or alvespimycin and GS-444217, significantly reduced the expression of these 3 inflammatory genes in diabetic kidneys at day 28 after IRI (Figure 6A–C). In contrast with the proinflammatory response, kidney gene expression of the anti-inflammatory marker *Arginase-1* was unaffected by diabetes, but was increased 20-fold at day 10 after IRI, which then declined to 3-fold at day 28 after IRI (Figure 6D). Senotherapy treatments had no effect on the gene expression of *Arginase-1* in diabetic kidneys at day 28 after IRI (Figure 6D).

### Senotherapy prevents progression of TIF in diabetic mice after IRI

**Figure 7:**
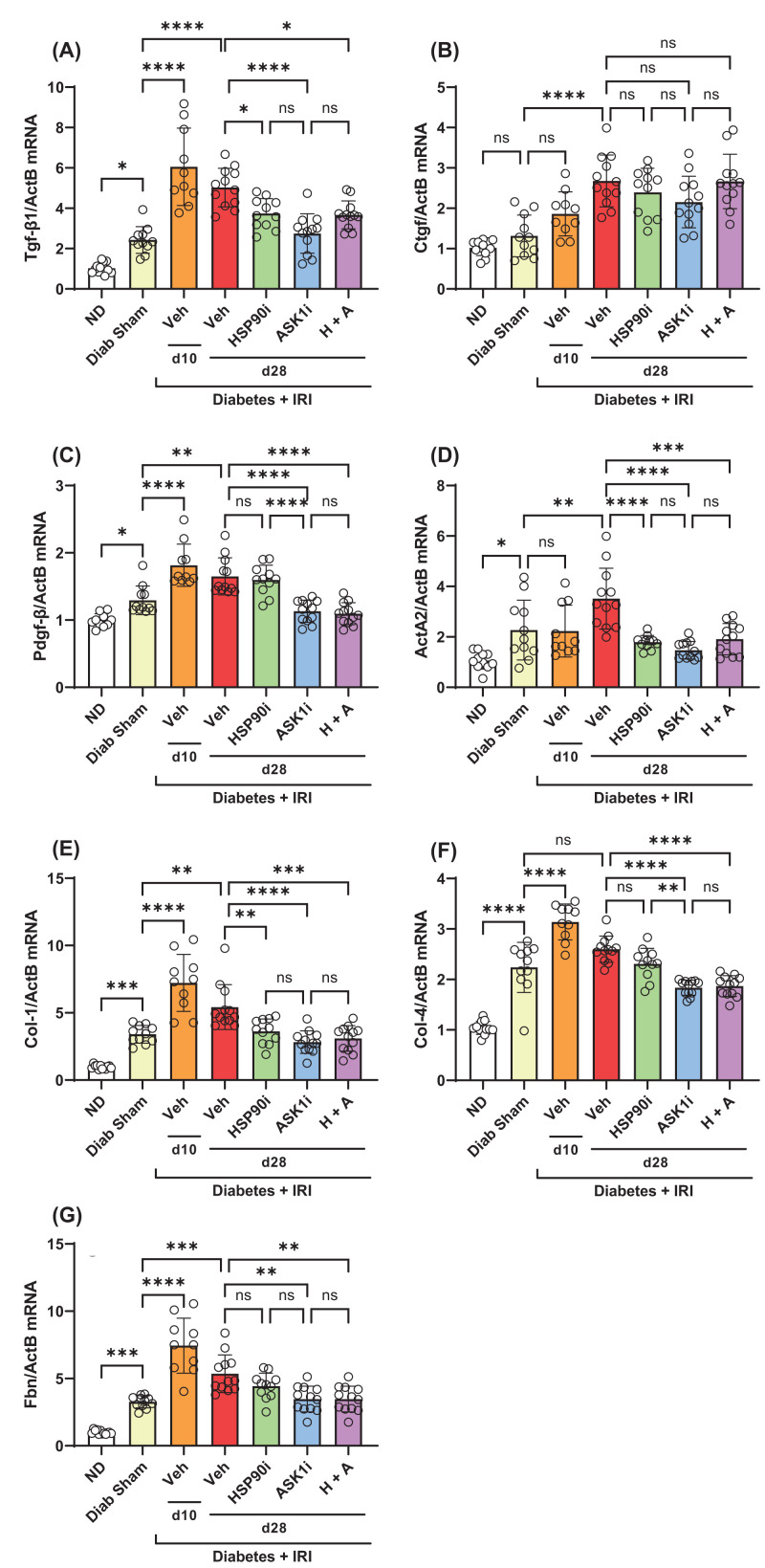
Senotherapies reduce fibrotic gene expression in diabetic mice with IRI Analysis of diabetic mouse kidneys at days 10 and 28 after IRI identified the impact of alvespimycin (HSP90i, green bars), GS-444217 (ASK1i, blue bars), and combined therapy (H+A, purple bars) on kidney gene expression of (**A**) *Tgf-β1* and (**B**) *Ctgf*, (**C**) *Pdgf-*β, (**D**) *ActA2*, (**E**) *Collagen I*, (**F**) *Collagen IV*, and (**G**) *Fibronectin* [*Fbn*]. Non-diabetic mice (ND, white bars), sham-operated diabetic mice (Diab Sham, yellow bars) and vehicle-treated mice with diabetes and IRI (Veh, red bars) were used as controls. Data = individual data points with mean ± SD; *n*=10–12; ****P<0.0001, ***P<0.001, **P<0.01, *P<0.05; ns = non-significant.

By week 12 of diabetes, we identified a 2- to 3-fold increase in the kidney gene expression of *Tgfb1*, but found no increase in *Ctgf/Ccn2* or *Pdgfb* mRNA levels (Figure 7A–C). In comparison, gene expression of each of these profibrotic cytokines was increased in diabetic kidneys at days 10 and 28 after IRI (Figure 7A–C). Treatment with alvespimycin alone, GS-444217 alone, or both, reduced gene expression of *Tgfb1*, but not *Ctgf*, in diabetic kidneys at day 28 after IRI (Figure 7A–C). However, only GS-444217 alone, or alvespimycin and GS-444217, reduced gene expression of *Pdgfb* in diabetic kidneys at day 28 after IRI (Figure 7A–C).

**Figure 8:**
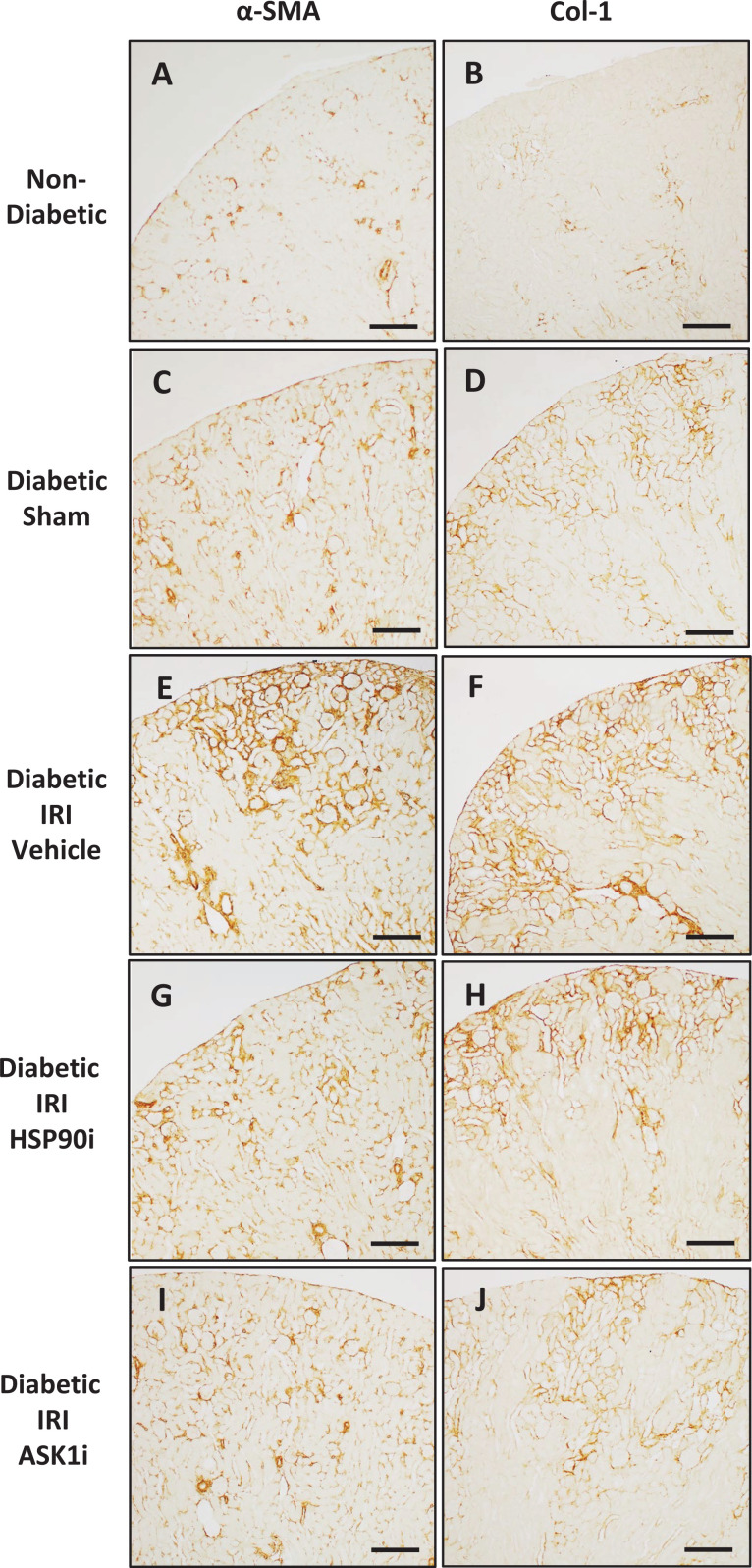
Senotherapies inhibit progression of TIF in diabetic mice with IRI Immunostaining shows expression of α-smooth muscle actin (α-SMA, brown) in the vessels of (**A**) a normal non-diabetic mouse kidney, and also in the interstitial myofibroblasts accumulating in (**C**) a diabetic mouse kidney at day 28 after sham surgery. The accrual of α-SMA+ interstitial myofibroblasts was substantially greater in (**E**) a diabetic mouse kidney at day 28 after IRI surgery, but was significantly reduced by treatment with (**G**) alvespimycin or (**I**) GS-444217. Similarly, deposition of collagen I (Col-1, brown) is seen around some vessels, glomeruli and tubules in (**B**) a normal non-diabetic mouse kidney, and also throughout the tubulointerstitium in (**D**) a mouse diabetic kidney at day 28 after sham surgery. Tubulointerstitial deposition of collagen-1 was more extensive in (**F**) a mouse diabetic kidney at day 28 after IRI surgery, but was significantly reduced by treatment with (**H**) alvespimycin or (**J**) GS-444217. Panels (A–J), bar = 200 µm.

**Figure 9:**
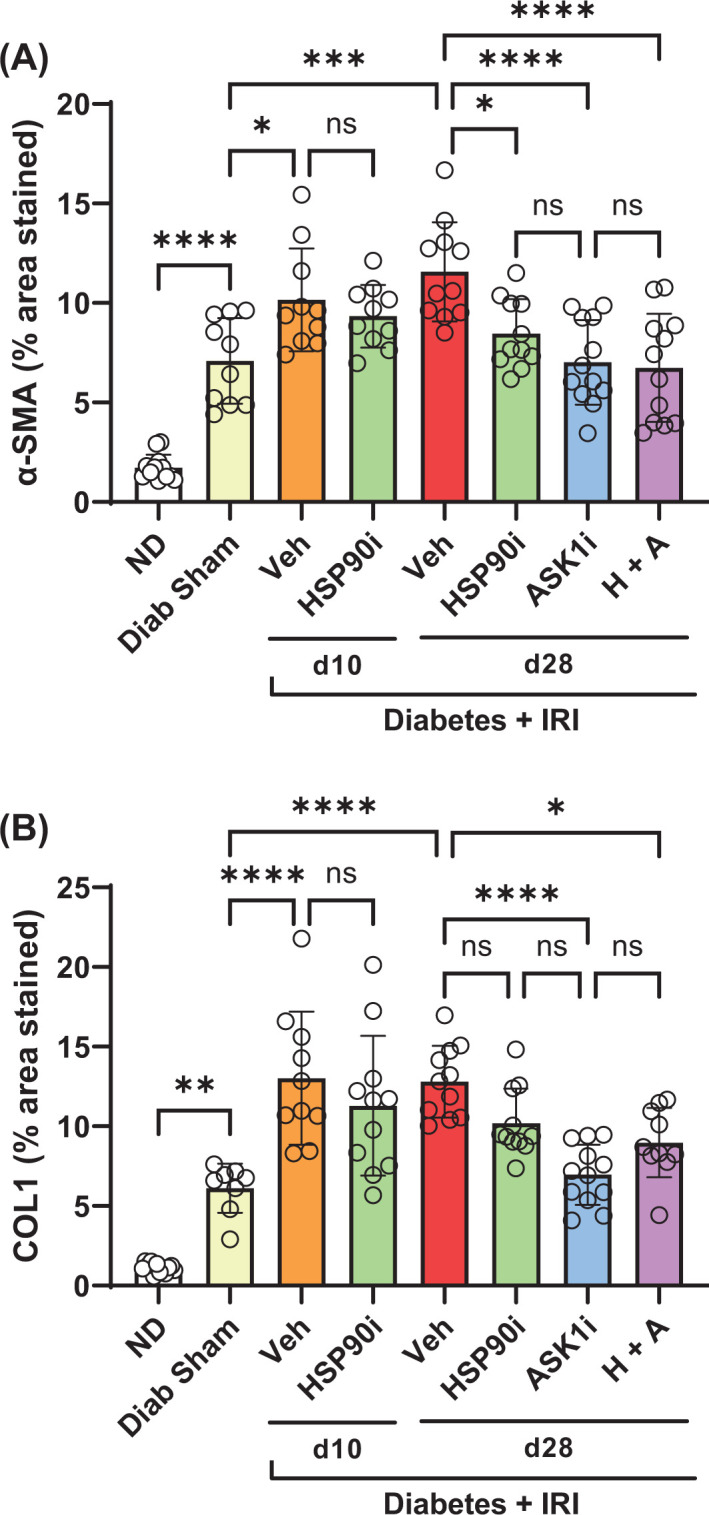
Analysis of the effects of senotherapies on TIF The impact of senotherapies on the tubulointerstitial deposition of (**A**) α-smooth muscle actin and (**B**) collagen 1 is shown graphically. In these graphs, treatments are displayed as alvespimycin (HSP90i, green bars), GS-444217 (ASK1i, blue bars), and combined therapy (H+A, purple bars). Non-diabetic mice (ND, white bars), sham-operated diabetic mice (Diab Sham, yellow bars) and vehicle-treated mice with diabetes and IRI (Veh, red bars) were used as controls. Data = individual data points with mean ± SD; *n*=10–12; ****P<0.0001, ***P<0.001, **P<0.01, *P<0.05; ns = non-significant.

Gene expression of the myofibroblast marker α-smooth muscle actin (*Acta2*) and matrix molecules collagen 1 (*Col1a1*), collagen IV (*Col4a3*) and fibronectin (*Fn1*) were all increased 2- to 3- fold in kidneys at week 12 of diabetes (Figure 7D–G). Applying IRI to diabetic mice resulted in further increases in the gene expression of *Acta2, Col1a1*, and *Fn1*, but not *Col4a3*, at day 28 after IRI (Figure 7D–G). Similarly, immunostaining analysis demonstrated accumulation of α-smooth muscle actin (α-SMA) and collagen I in the interstitium of diabetic kidneys at day 28, which was substantially increased after IRI (Figure 8A–F). Treatment with alvespimycin alone reduced both mRNA and protein levels of α-SMA and Collagen I in diabetic kidneys with IRI (Figures 7D,E and 8G,H and 9A,B). However, treatment with GS-444217 alone, or alvespimycin and GS-444217, provided a more substantial protection against fibrosis by reducing the kidney gene expression of *Col4a3* and *Fn1* (Figure 7F,G) in addition to inhibiting kidney levels of α-SMA and collagen 1 (Figures 8E–J and 9A,B).

## Discussion

In the present study, we demonstrated that intervention with a senolytic and/or senostatic therapy can inhibit the accelerated progression of DKD induced by a subsequent episode of AKI. Notably, the vast majority of senescent cells in these kidneys were found to be TECs, suggesting that tubular senescence plays a critical role in the progression of tubulointerstitial damage in DKD, which is supported by other preclinical studies [5,29].

Moderate IRI resulted in acute tubular damage on day 3 in diabetic kidneys which remained unresolved by day 28, consistent with previous findings in this model [6,7]. Similarly, we found that urine excretion of the senescence marker Cdkn1a was acutely elevated after IRI, but returned to near pre-IRI levels by day 28. However, analysis of kidney gene expression and immunostaining showed that the increased tubular senescence which accompanied IRI tubular injury persisted at day 28, which was associated with accumulation of macrophages, myofibroblasts and collagen in the tubulointerstitium. Kidney production of SASPs which promote macrophage recruitment (*Ccl2*), macrophage activation (*Tnf*), fibroblast growth (*Pdgfb*), and extracellular matrix production (*Tgfb1*), also remained elevated at day 28, providing a mechanistic basis for the observed chronic inflammation and TIF.

While a pulse treatment with a senolytic drug (alvespimycin) on days 3, 6, and 9 reduced tubular senescence, inflammation, myofibroblasts and TIF at day 28, ongoing intervention treatment with a senostatic drug (GS-444217) provided greater benefit, which included protection from renal function impairment (increased BUN) and reduced kidney production of some SASPs (*Tnf, Ccl2, Pdgfb*). The therapeutic effect of alvespimycin was found to be delayed with no protection seen at the end of the treatment (day 10), but with significant protection being observed at day 28. In comparison, intervention with GS-444217 from days 10 to 28 provided similar protection from tubular senescence, but increased protection from kidney production of SASPs, which was similar when both treatments were combined. This suggests that inhibition of SASPs is important for protecting renal function, which is supported by previous studies where GS-444217 inhibited kidney production of these cytokines and renal function impairment in diabetic models [21,30], although senescence was not investigated in these studies.

In our disease model, immunodetection of CDKN1A+ TEC, in contrast with kidney mRNA levels of *Cdkn1a* or *Cdkn2a*, decreased over time after an acute IRI response, possibly due to antigen shedding by injured tubules or immune clearance of senescent TEC. This may help explain why it was difficult to identify the removal of senescent TEC by the senolytic therapy alvespimycin (HSP90 inhibitor). While alvespimycin has been shown to be selectively cytotoxic to senescent cells *in vitro* [13], it is less clear how this senolytic effect proceeds *in vivo*. Previous research [13] has shown that the dosage regimen of alvespimycin used in this study was able to substantially reduce the *Cdkn2a* senescence marker in a mouse model of accelerated senescence (Ercc1-/Δ mice) at 4 days after completion of treatment. This suggests that the senolytic effect of alvespimycin *in vivo* occurs after gradual accumulation in senescent cells reaches toxic levels over time, which is consistent with the delayed therapeutic benefits observed in our study.

Notably, interventions with alvespimycin and GS-444217 were given sequentially, and not together, because we wanted to avoid the possibility that ASK1 inhibition might prevent HSP90 inhibitor-induced death of senescent cells. While our results indicate that giving alvespimycin and GS-444217 sequentially had similar benefit to treatment with GS-444217 alone, it does not rule out the possibility that administering both drugs together could have added benefit. However, given the delayed therapeutic effect of alvespimycin, identifying a benefit from administering both drugs together may require a longer period of intervention beyond day 28.

Another novel finding from this study is that intervention with an ASK1 inhibitor protects against the progression of DKD by reducing tubular senescence. Previously, it was known that ASK1 inhibition protects against oxidative stress, inflammation and fibrosis in diabetic kidneys, which could reduce the progression of renal function impairment in diabetic patients [19,28]. The current study demonstrates that these therapeutic effects are at least partly due to a reduction in tubular senescence and production of SASPs, thereby providing new mechanistic insight into how ASK1 inhibitors protect against DKD and possibly other types of tissue injury.

Recent animal model studies have indicated that other molecular mechanisms such as complement activation of the C5a receptor and pentraxin 3-induced mitochondrial dysfunction play a role in the development of senescence in kidney tubules in models of CKD involving diabetes or ischemic reperfusion injury [31,32]. These mechanisms were not examined in our evaluation of senotherapies in mice with diabetes and IRI. However, it is feasible that these mechanisms may be regulated by ASK1 and may play a role in the reduction of tubular senescence associated with ASK1 inhibition.

In conclusion, we have demonstrated that superimposing AKI on diabetic kidneys can promote TIF and renal dysfunction by inducing increased tubular senescence. Furthermore, we have shown that a senolytic HSP90 inhibitor and a senostatic ASK1 inhibitor can be effective in suppressing the progression of DKD associated with an episode of AKI. The clinical relevance of this finding is supported by the knowledge that the incidence of end stage renal disease (ESRD) increases in diabetic patients in proportion to the number of episodes of AKI and their severity [10]. Applying senotherapies to patients with DKD, particularly those who experience AKI, may significantly reduce the progression to ESRD. Hopefully, future studies will identify more effective and selective strategies for reducing tubular senescence in DKD.

## Clinical perspectives

- Clinical research indicates that the progression of DKD, leading to TIF and renal function impairment, is dependent on episodes of AKI.
- Using an animal model to superimpose mild acute kidney injury on pre-existing diabetic kidney injury, we established that the resulting renal damage promotes tubular senescence and that intervention with senotherapies can suppress the development of tubulointerstitial fibrosis and renal function impairment.
- This study establishes that targeting tubular senescence is critical for preventing the progression of diabetic kidney disease and identifies inhibition of ASK1 as a novel and clinically applicable strategy to achieve this outcome in patients.

## Supplementary Material

Supplementary Figures S1-S2 and Table S1

## Data Availability

All authors agree to make to make any materials, data, and associated protocols available upon request. Data from individual samples are displayed in most figures.

## Open Access

Open access for this article was enabled by the participation of Monash University in an all-inclusive *Read & Publish* agreement with Portland Press and the Biochemical Society under a transformative agreement with CAUL.

## Abbreviations

α-SMA: α-smooth muscle actin
ASK1: apoptosis signal-regulating kinase-1
AKI: acute kidney injury
BUN: blood urea nitrogen
CKD: chronic kidney disease
DKD: diabetic kidney disease
ESRD: end stage renal disease
HSP: heat shock protein
IRI: ischemia–reperfusion injury
SASP: senescent-associated secretory protein
TEC: tubular epithelial cell
TIF: tubulointerstitial fibrosis
