# Supplementary material for: Intervention treatment reducing cellular senescence inhibits tubulointerstitial fibrosis in diabetic mice following acute kidney injury
Source: Clin Sci (Lond). 2024 Mar 5;138(5):309–26. doi: 10.1042/CS20231698 (PMC10914710; doi:10.1042/CS20231698)
Supplement: Supplementary Figures S1-S2 and Table S1 [file CS-2023-1698_supp.pdf]

Supplementary Figure 1: Effect of Diabetes and IRI on Body Weight.

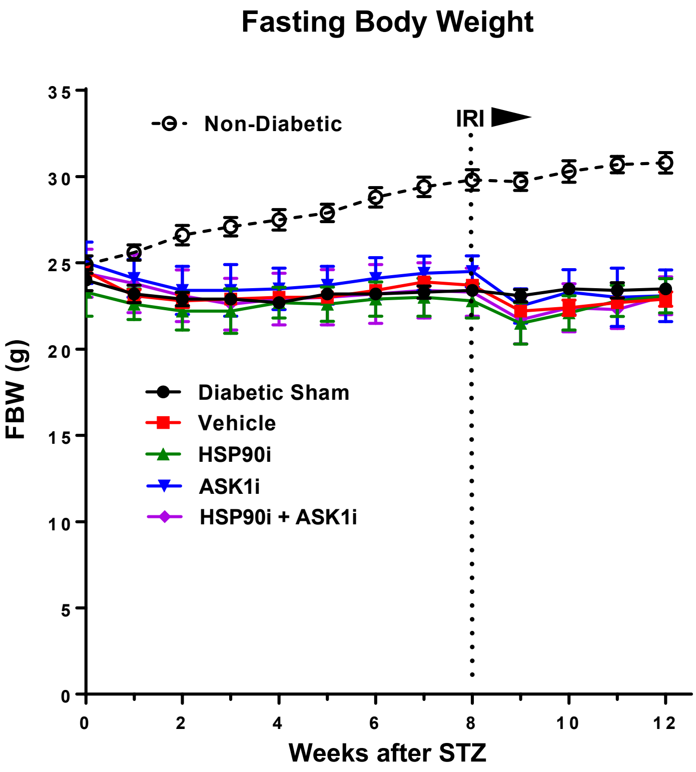

Supplementary Fig. 1. Graphs show the 3h fasting body weight of diabetic and non-diabetic mice taken at weekly intervals during experimentation. The dotted vertical line indicates when IRI or sham surgery was performed on diabetic mice.
Data = mean ± SD. n=10-12.

Supplementary Figure 2: Effect of Alvespimycin treatment on liver function.

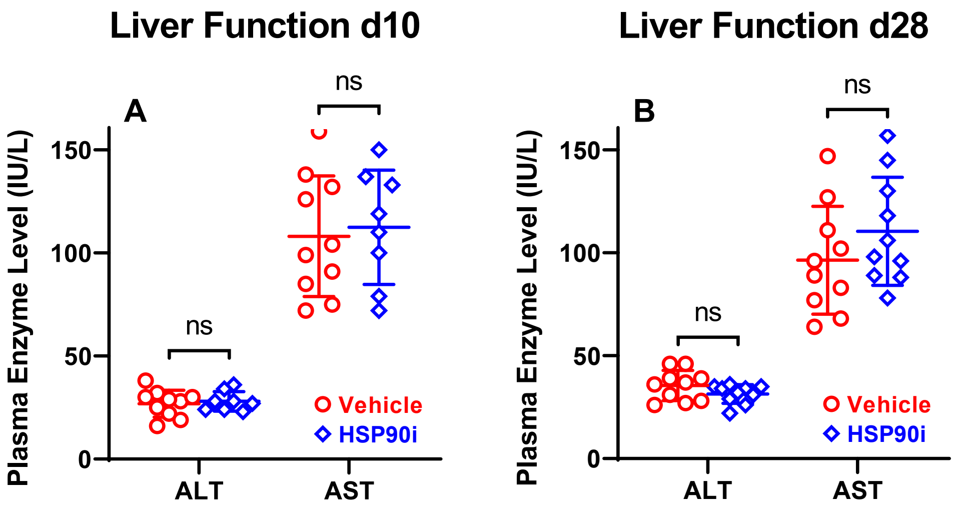

Supplementary Fig. 2. Graphs show that administration of alvespimycin (HSP90i, 10mg/kg on days 3, 6 and 9 after IRI) has no effect on blood levels of aspartate transaminase [AST] and alanine transaminase [ALT] at (A) day 10 and (B) day 28 after IRI compared to vehicle alone. Data = individual data points with mean ± SD. n=8-10. ns = non-significant.

Supplementary Table 1: Commercial mouse gene expression assays

(Applied Biosystems, Thermo-Fisher Scientific, USA)

**Target gene**	**Taqman™ Gene Expression Assay ID**
Acta2/αSMA	Mm00725412_s1
Arginase-1	Mm0475899_m1
Ccl2/Mcp1	Mm00441242_m1
Cd68	Mm03047343_m1
Cdkn1a	Mm04205640_g1
Cdkn2a	Mm00494449_m1
Collagen 1a1	Mm00801666_g1
Collagen 4a1	Mm01210125_m1
Ctgf	Mm01192933_m1
Fibronectin	Mm01256744_m1
Kim1/Havcr1	Mm00506686_m1
Klotho	Mm00502002_m1
Pdgf-β	Mm00440677_m1
Tnf-α	Mm00443258_m1
Tgf-β1	Mm01178820_m1
